# Neuromuscular Effects of Common Krait (*Bungarus caeruleus*) Envenoming in Sri Lanka

**DOI:** 10.1371/journal.pntd.0004368

**Published:** 2016-02-01

**Authors:** Anjana Silva, Kalana Maduwage, Michael Sedgwick, Senaka Pilapitiya, Prasanna Weerawansa, Niroshana J. Dahanayaka, Nicholas A. Buckley, Christopher Johnston, Sisira Siribaddana, Geoffrey K. Isbister

**Affiliations:** 1 Monash Venom Group, Department of Pharmacology, Monash University, Clayton, Victoria, Australia; 2 Faculty of Medicine and Allied Sciences, Rajarata University of Sri Lanka, Saliyapura, Sri Lanka; 3 South Asian Clinical Toxicology Research Collaboration, University of Peradeniya, Peradeniya, Sri Lanka; 4 Clinical Toxicology Research Group, University of Newcastle, New South Wales, Australia; 5 Clinical Pharmacology, University of Sydney, Sydney, Australia; Institut de Recherche pour le Développement, BENIN

## Abstract

**Objective:**

We aimed to investigate neurophysiological and clinical effects of common krait envenoming, including the time course and treatment response.

**Methodology:**

Patients with definite common krait (*Bungarus caeruleus*) bites were recruited from a Sri Lankan hospital. All patients had serial neurological examinations and stimulated concentric needle single-fibre electromyography (sfEMG) of orbicularis oculi in hospital at 6wk and 6–9mth post-bite.

**Principal Findings:**

There were 33 patients enrolled (median age 35y; 24 males). Eight did not develop neurotoxicity and had normal sfEMG. Eight had mild neurotoxicity with ptosis, normal sfEMG; six received antivenom and all recovered within 20–32h. Seventeen patients developed severe neurotoxicity with rapidly descending paralysis, from ptosis to complete ophthalmoplegia, facial, bulbar and neck weakness. All 17 received Indian polyvalent antivenom a median 3.5h post-bite (2.8–7.2h), which cleared unbound venom from blood. Despite this, the paralysis worsened requiring intubation and ventilation within 7h post-bite. sfEMG showed markedly increased jitter and neuromuscular blocks within 12h. sfEMG abnormalities gradually improved over 24h, corresponding with clinical recovery. Muscle recovery occurred in ascending order. Myotoxicity was not evident, clinically or biochemically, in any of the patients. Patients were extubated a median 96h post-bite (54–216h). On discharge, median 8 days (4–12days) post-bite, patients were clinically normal but had mild sfEMG abnormalities which persisted at 6wk post-bite. There were no clinical or neurophysiological abnormalities at 6–9mth.

**Conclusions:**

Common krait envenoming causes rapid onset severe neuromuscular paralysis which takes days to recover clinically consistent with sfEMG. Subclinical neuromuscular dysfunction lasts weeks but was not permanent. Antivenom effectively cleared venom but did not prevent worsening or reverse neuromuscular paralysis.

## Author Summary

Common krait bites cause muscular paralysis due to the venom disrupting communication between the nerves and muscles. This becomes life-threatening for the patient if there is paralysis of the muscles used for breathing. We studied the severity of paralysis, long term effects and the value of antivenom treatment in authenticated Indian krait bite patients from Sri Lanka. In addition to standard treatment with antivenom, the patients had single-fibre electromyography done, a sensitive neurophysiological test that detects the abnormalities of communication between the nerves and muscles. Half of the patients had severe paralysis and required mechanical ventilation, and the remainder had mild or no effects. Antivenom was given to all patients with severe paralysis and most with mild effects. However, despite antivenom binding all free venom after it was administered, it did not prevent or reverse already developed paralysis. Clinically evident paralysis resolved after a few days, but the neurophysiological abnormalities lasted for weeks. No permanent neurological damages were noted at 6 to 9 months after the snake bite.

## Introduction

Globally snake envenoming is a major cause of morbidity and mortality in the rural tropics [1]. Neurotoxicity causing paralysis is one of the major clinical syndromes of snake envenoming, and occurs mainly with elapids (Australasian elapids, American coral snakes, Asian kraits and some cobra species)[2,3].Envenoming may result in prolonged hospital stay if ventilatory support is available or significant mortality where such medical resources are not available. Despite the magnitude of the health impact of neurotoxic snake envenoming, it continues to be associated with several unresolved issues of clinical importance[2].

Envenoming due to krait (Genus: *Bungarus*) bites is a common, serious health issue in South and South-East Asia. Common krait (*Bungarus caeruleus*) is distributed throughout South Asia, and is responsible for large numbers of cases of severe neurotoxic envenoming each year[4]. It results in a descending flaccid paralysis progressing to life threatening respiratory paralysis unless mechanical ventilation is available[5–7]. Krait bites typically occur at night and are not painful, so many patients do not notice the bite and continue sleeping[7–10],which delays medical care.

Neuromuscular paralysis in krait envenoming is characterized by progressive descending paralysis. Krait venom contains β-bungarotoxins, which are presynaptic neurotoxins with phospholipase A_2_ activity and considered to be the major cause of paralysis[11]. The pre-synaptic action is irreversible and is the reason that once paralysis develops it is not reversed with antivenom[12].

The pathophysiology of neuromuscular paralysis in snake envenoming remains poorly understood. This is due to the lack of detailed clinical studies of authenticated bites that report the time course of paralysis, including the recovery of paralysis, and very few studies of neurophysiological function in snake envenoming[2]. There is also little evidence for the effectiveness of antivenom in reversing neuromuscular paralysis[13,14]. Neurophysiological testing could provide objective evidence of the progression and recovery of neurotoxicity, and the response to antivenom. Previous neurophysiological testing in krait bites with nerve conduction studies and repetitive nerve stimulation tests, provide some information on krait neurotoxicity[15]. Single fibre electro-myography (sfEMG) is a more sensitive test of neuromuscular function[16]. It has only been reported in one study of neuromuscular paralysis from Papuan taipan envenoming.[17]Better methods to measure neuromuscular dysfunction in neurotoxic envenoming will improve our understanding of neuromuscular paralysis and the clinical benefit of antivenom.

This study aimed to describe detailed neurological examinations in patients with krait envenoming, the use sfEMG to objectively measure the time related progression and recovery of neuromuscular paralysis and the relationship to serum venom concentrations.

## Materials and Methods

### Ethics statement

Ethical approval was granted by the research ethics review committees of the University of Peradeniya, Sri Lanka, the Rajarata University of Sri Lanka, and Monash University, Australia. Written informed consent was sought prior to the recruitment of all patients. For patients aged 16 and 17 years consent was also obtained from the patient’s parent or guardian.

### Study setting and the patients

This study is part of a prospective study of all snakebites admitted from April to October 2014 to the Teaching Hospital, Anuradhapura, a tertiary care centre and the largest hospital in the north central province of Sri Lanka. The region has one of the highest incidences of snakebite in Sri Lanka.

**Fig 1:**
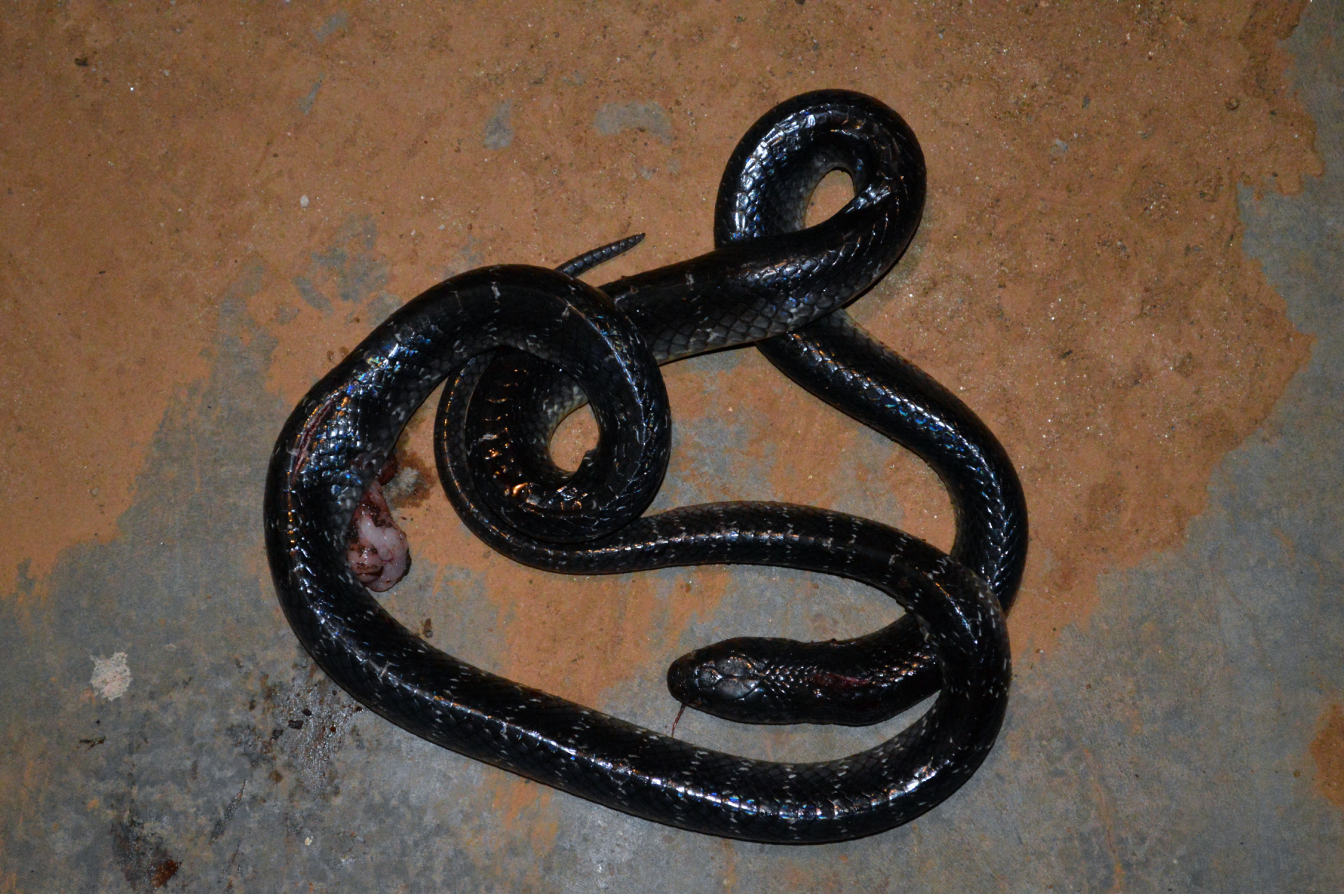
Common krait. An adult common krait (Bungarus caeruleus) specimen, 92.5 cm in total length, that caused severe neuromuscular paralysis.

Patients aged 16 years or over with definite common krait (*B*. *caeruleus*) bites were recruited. Cases were authenticated as common krait bites by either expert snake identification or detection of krait venom in patient serum. All patients presenting with live or dead specimens of the offending snakes (Fig 1A), had the snake identified by one author (AS) who is a herpetologist. Patients with clinical suspicion of a common krait bite had serum tested for krait venom by venom-specific enzyme-immunoassay.

### Data collection

All patients with suspected krait envenoming had a complete neurological examination on admission to hospital, then repeat examinations every 2h for the first 24h post-bite, and then every 4h for the remainder of their hospital stay. After discharge patients had a full neurological examination at six weeks and six months post-bite to detect residual neurological impairment. The Medical Research Council scale for muscle strength[18] was used wherever appropriate. For the assessment of respiratory muscles, tidal volume was measured using a spirometer. Ptosis was graded from grade I to III using a visual analogue scale, with complete ptosis being grade III. Weak or sluggish eye movements in one or all directions was considered to be partial ophthalmoplegia and absent eye movements in all directions, complete ophthalmoplegia. At each time point patients were specifically examined for features of autonomic neurotoxicity (heart rate, blood pressure, lacrimation, sweating and salivation), central effects (level of consciousness and occulocephalic reflexes) and myotoxic effects (muscle pain and tenderness, both local and general).

Clinical assessments were done by one author (AS) or medically qualified clinical research assistants. All assessments performed by clinical research assistants were reviewed by AS and approximately one third were reviewed by another medically trained author (SS). In addition to a neurological assessment, all patients had a full clinical examination on admission, at 12h and 24h post-bite, and then daily until discharged. Local effects included pain, swelling, paraesthesia or regional lymphadenopathy and non-specific systemic effects were defined as headache, nausea, vomiting or abdominal pain. All assessments were recorded using a pre-formatted clinical data form.

Antivenom administration was decided by the treating physician. All patients who received antivenom in this study received 20 vials of Indian polyvalent antivenom as the first dose from VINS Bioproducts (batch numbers: 01AS11119, 01AS11121, 01AS13100, 01AS14025, 01AS14026, 01AS14035). No patient in this study received a second antivenom dose. The antivenom infusion was ceased briefly for 5–10min in patients who developed anaphylaxis. Antivenom reactions were treated according to the attending physician with adrenaline, antihistamines and corticosteroids.

During review visits patients had a routine physical and neurological examination. They were also questioned specifically about the presence of neuromuscular effects that prevented them performing routine daily work, and about any recovery of local effects.

Patients were classified into three groups based on the presence and severity of neurotoxicity. The first group included patients who developed no clinical evidence of neurotoxicity. The second group was patients with mild neurotoxicity defined as the presence of one of the following clinical features; ptosis, ophthalmoplegia or facial muscle weakness, but not bulbar, respiratory or limb weakness. The third group was severe neurotoxicity defined as patients developing paralysis that involved bulbar and respiratory muscles requiring mechanical ventilation.

### Single-fibre electromyography (sfEMG)

Stimulated sfEMG of the orbicularis oculi muscle was performed using disposable concentric needle electrodes (diameter: 0.3mm) and a portable Medelec Synergy N2 EMG system (Medelec Synergy, UK). All tests were performed by AS at the bedside during the hospital admission and in a separate examination room during review visits at 6 weeks and 6 months. In order to establish the baseline values for stimulated sfEMG jitter for normal Sri Lankan subjects, 29 healthy individuals had sfEMG performed under the same conditions. The normal upper limits of jitter for individual fibres and individual subjects were established as 30μs and 20.6μs respectively[19].

Stimulation of the suprazygomatic branches of the facial nerve was by monopolar needle electrode at a frequency of 10Hz, with the stimulus delivered as rectangular pulses of 0.1ms. Stimulation intensity was increased until the appearance of visually discernible twitches of the orbital part of the orbicularis oculi muscle. Stimulus strength did not exceed 2mA. All recordings were done after the stimulus intensity had reached supra-threshold for the fibre being examined. Recording and analysis of the sfEMG followed Kouyoumdjian and Stålberg[20] and the jitter of individual fibres was expressed as a mean consecutive difference (MCD). Neuromuscular block was where there was no transmission across the neuromuscular junction in fibres on the sfEMG recording.

### Common krait venom specific enzyme immunoassay

Blood samples from patients with suspected krait bites were collected on admission and at 1, 4, 8, 12 and 24h post-bite, and daily thereafter. All samples were immediately centrifuged, and serum aliquoted and frozen at -80°C. Venom was quantified using a sandwich enzyme immunoassay as previously described[21–23]. In brief, rabbit IgG antibodies (provided by University of Rajarata) were bound to microplates as well as conjugated to biotin for the sandwich enzyme immunoassay, detecting with streptavidin-horseradish peroxidase. The lower limit of detection for the assay was 0.2ng/ml.

### Serum creatinine kinase assay

Creatine kinase (CK) activity was measured in the serum samples of patients obtained 24h post-bite, or a close to this time point as possible if the 24h sample was unavailable. Creatine kinase was measured using a commercially available assay kit using the CK-NAC Reageant (Creatine Kinase, activated by N-acetylcysteine, Thermo Scientific, Middletown, VA, USA).

### Analysis

Analysis of clinical and sfEMG data was done using Prism, version 6.05 (GraphPad Software, Inc.). Continuous variables were reported as medians, range and interquartile range (IQR). Jitter values and creatine kinase concentrations of different patient groups were analysed using one-way ANOVA followed by multiple comparison tests.

## Results

During the study period 773 snakebite patients were admitted and 38 of these were suspected common krait bites. Thirty one patients had the offending snake positively identified as a common krait and two others were positive for krait venom in their blood. The remaining five cases were only suspected common krait bites based on circumstances of the bite, but had no features of local or systemic envenoming, despite having fang marks. None of these patients had a previous history of neurological disorders or therapeutic agents known to cause neurotransmission abnormalities. Table 1 provides the demographic features of the 33 confirmed cases of common krait bites.

**Table 1:** Demographic features of 33 definite Indian Krait bite patients.

Age (median, range)	35	16–78
Sex (male)	24	73%
Occupation (Farmer)	22	67%
(manual laborer)	5	15%
(security personnel)	2	6%
(student)	2	6%
(Other)	2	6%
Bite time (night: 1900–0600 hrs)	30	91%
Activity (Sleeping on the floor)	22	67%
(Sleeping on the bed)	3	9%
(household work)	4	12%
(Other)	4	12%
Place (home)	25	76%
(hut in the farmland)	4	12%
(Home garden)	2	6%
(other)	2	6%
Site of bite (lower limbs)	12	36%
(upper limbs)	12	36%
(Trunk)	4	12%
(head)	1	3%
(site uncertain)	4	12%
Under influence of alcohol when snake bite took place	4	12%
Distance from the place of bite to the nearest hospital (median, range; in km)	6	1.5–20
Mode of transport used to reach the nearest hospital (motorbike)	17	52%
(Three wheeler)	10	30%
(other)	6	18%
Time since bite to reach nearest hospital (median, range; in hours)	0.87	0.16–4.5
Time since bite to reach the study hospital (median, range; in hours)	3.3	1.5–7.0
First aid and home remedies (washed the bite site)	3	9%
(local application of lime juice)	3	9%
(application of tourniquet proximal to the bite site)	3	9%
(herbal decoctions)	3	9%
Previous history of snakebites	3	9%
Total length of the offending snake (median, range; in cm)	90.6	32.2–122.2
Sex of the offending snake (female)	15	58%

### Clinical effects during hospital stay

Eight patients who brought the offending snake and had evidence of a bite with fang marks, did not develop neurotoxic features during their hospital stay. None of these received antivenom (Table 2).

**Table 2:** Comparison of the clinical effects and treatment of the patients who developed no neurotoxicity, mild neurotoxicity and severe neurotoxicity following common krait envenoming.

	No neurotoxicity	Mild neurotoxicity	Severe neurotoxicity
Number of patients	8	8	17
Hours from bite to hospitalization: median (range)	3.0 (1.5–6.0)	3.0 (1.0–6.0)	3.3 (1.5–7.0)
Days of hospital stay: median (range)	2 (1–3)	3 (2–3)	8 (4–12)
**Neurotoxicity**			
Maximum ptosis observed			
• Partial	-	7 (87.5%)	5 (29.4%)
• Complete	-	1 (12.5%)	12 (70.6%)
Maximum ophthalmoplegia observed			
• Partial	-	1 (12.5%)	3 (17.6%)
• Complete	-	0	14 (82.4%)
Strabismus	-	-	11 (64.7%)
Facial weakness	-	1 (12.5%)	17 (100%)
Neck flexion weakness (power <5)	-	-	15 (88.2%)
Difficulty in swallowing	-	-	17 (100%)
Low pitched voice	-	-	12 (70.6%)
Tidal volume <250ml	-	-	17 (100%)
Reduced upper limb power (Power <5)	-	-	12 (70.6%)
Reduced lower limb power (power <5)	-	-	5 (29.4%)
Diminished or absent deep tendon reflexes			9 (52.9%)
Intubation and mechanical ventilation	-	-	17 (100%)
Autonomic features	-	-	6 (35.3%)
**Other features of envenoming**			
Generalized myalgia	3 (32.7%)	2 (25%)	7 (41.2%)
Generalized muscle tenderness	2 (25%)	2 (25%)	7 (41.2%)
Local envenoming	5 (62.5%)	6 (75%)	9 (52.9%)
Non-specific systemic envenoming	-	5 (62.5%)	13 (76.5%)
**Treatment**			
Antivenom given	-	6 (75%)	17 (100%)
Hours from bite to antivenom: median(range)	-	7.5 (2.8–13.0)	3.5 (2.8–7.2)
Adverse reactions to antivenom	-	6 (100%)	13 (76.5%)

Eight patients had mild neurotoxicity and six of these received antivenom (Table 2). All these patients had partial ptosis on admission. In one patient, this progressed to complete ptosis with partial ophthalmoplegia and mild facial weakness within 12h of the bite, but 2h after antivenom was given. Two patients not receiving antivenom did not have any progression of neurotoxicity. All eight patients were fully recovered the next day and were discharged 20 to 32h post-admission.

**Fig 2:**
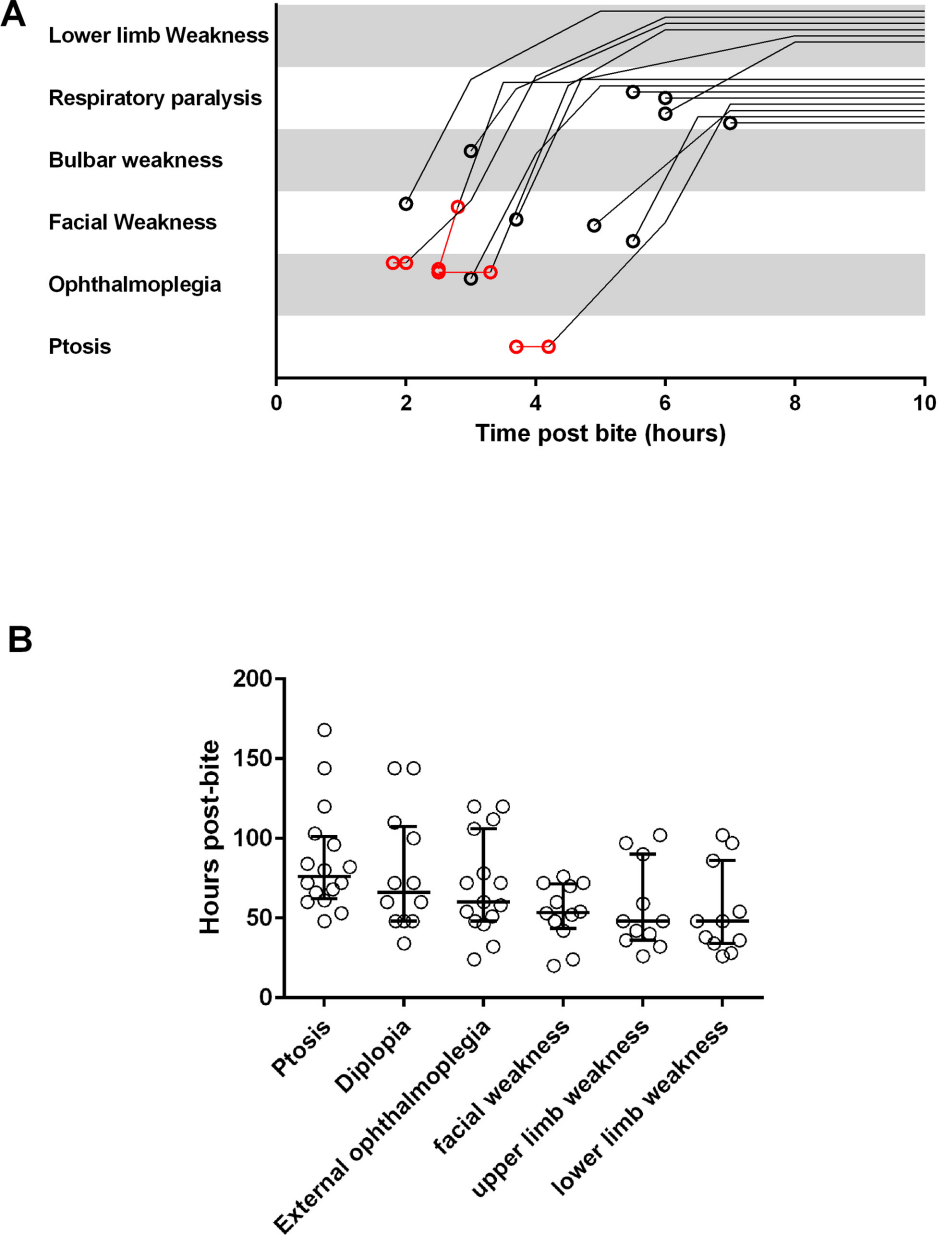
Time related change of neuromuscular paralysis. **A,** Noodle plot showing the evolution of paralysis in 14 patients with severe neurotoxicity. Note the pre-antivenom events are in red; **B,** Scatter plots of the time taken for the complete resolution of five major clinical effects of neurotoxicity (muscle groups), including ptosis, diplopia, external ophthalmoplegia, facial weakness, upper limb weakness and lower limb weakness in the 17 patients with severe neuromuscular paralysis.

Seventeen patients developed severe neurotoxicity with a range of neurotoxic clinical features on admission (Table 2). Three patients appeared to have altered consciousness and had no respiratory effort on admission with oxygen saturations < 50%. These three were immediately intubated and ventilated. All 17 patients were given antivenom a median of 3.5h post-bite (2.8–7.2h). Antivenom therapy did not stop progression of neurotoxic features in any patient and intubation and mechanical ventilation was required within 7h of the snake bite in all 17 patients. The clinical features of neurotoxicity progressed in a descending order from eyes to peripheral limbs (Fig 2A). Five patients developed complete limb paralysis with areflexia occurring within 8h of the bite (Figs 1C and 2A).

Neuromuscular function began to recover in all 17 patients on average 30h post-bite (22–51h). Muscle groups recovered in the reverse order to that in which paralysis developed. Fig 2B shows the time scale of the recovery of neurotoxic features. Patients were extubated a median of 96h post-bite (54–216 h).

One patient with severe paralysis and no motor function appeared to be in a deep coma with absent brainstem reflexes 12h post-bite. There was no recordable response on sfEMG done 10h and 16h post-bite. The patient had slight movement of the toes in response to a sternal rub 26h post-bite, but no occulocephalic reflex. The occulocephalic reflex recovered 6h later, followed by gradual ascending recovery of all motor function and sfEMG jitter.

All 33 patients were discharged with no clinically detectable neuromuscular paralysis. However, on discharge 23 patients had no pain, touch or temperature sensation in an area of 3–8cm diameter around the bite site. Of 23 patients who received antivenom, 19 developed an adverse reaction and six of these developed severe anaphylaxis with hypotension (systolic blood pressure <90mmHg) and tissue hypoxia (SpO_2_<92%) that required resuscitation. Of these, three were intubated for the antivenom reaction before they developed respiratory muscle paralysis due to envenoming.

### Clinical effects: Long term

At 6 weeks, six of eight patients with no acute neurotoxicity, seven of eight with mild neurotoxicity and 14 of 17 with severe neurotoxicity were reviewed. None had clinically detectable neuromuscular paralysis. Sixteen patients (five mild and 11 severe neurotoxicity) still had absent pain, touch and temperature sensation around the bite site (4–7cm diameter area).

At 6 to 9 months after the krait bite, six with no acute neurotoxicity, five with mild neurotoxicity and ten with severe neurotoxicity were reviewed and there was no evidence of clinical neuromuscular dysfunction. All sensory abnormalities around the bite site had resolved and the patients stated the feeling of numbness around the bite site disappeared 2 to 3 months post-bite.

### sfEMG

**Fig 3:**
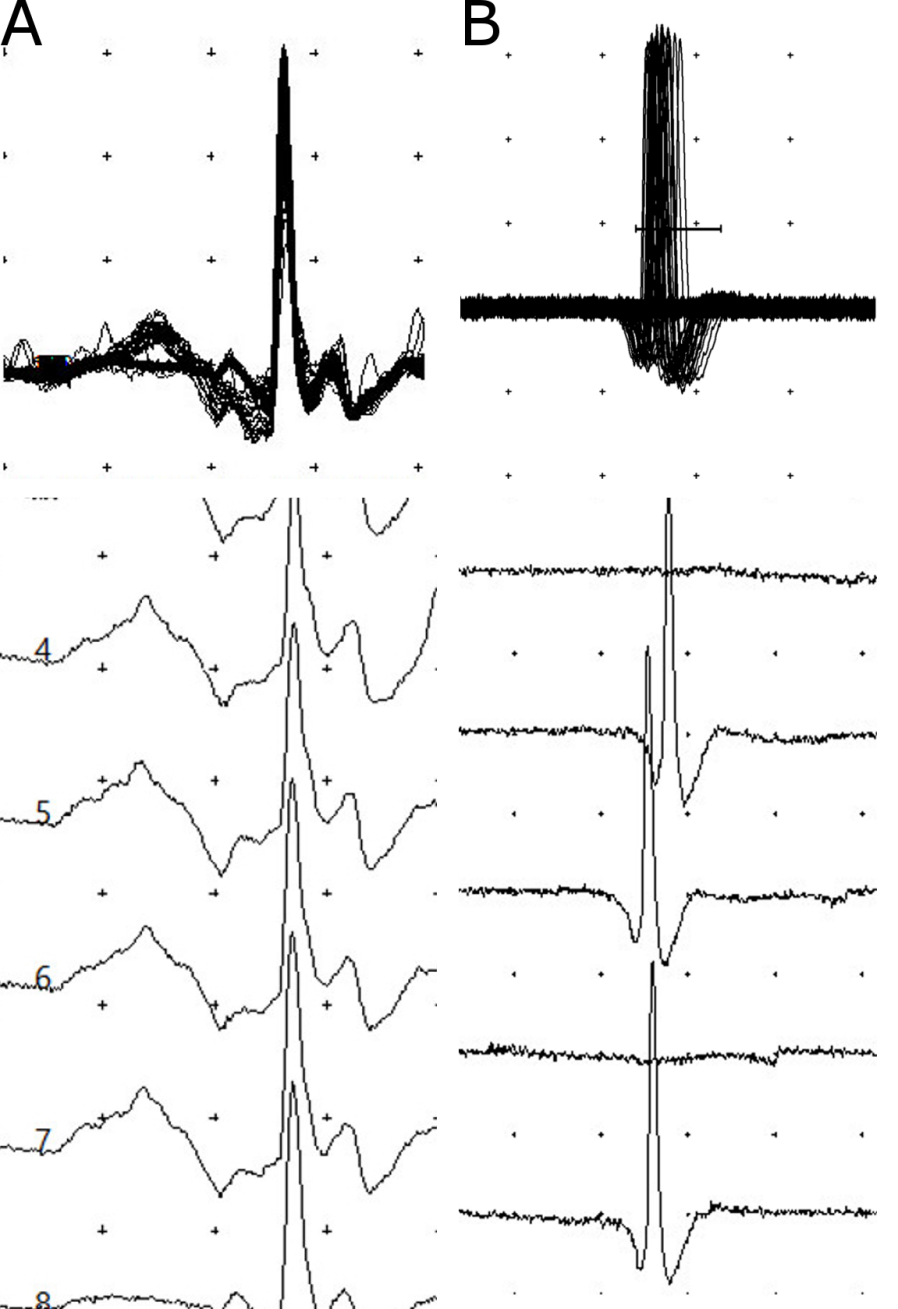
Superimposed and non-superimposed sfEMG recordings of the orbicularis oculi muscle of patients following krait bites. **A,** recordings of a patient on admission with no neurotoxicity indicating the normal jitter (14.5μs) and no blocks; **B,** high jitter (61.6μs) with intermittent blocks seen in a patient on admission with severe neuromuscular paralysis. (The distance between two dots represents 200μV vertically and 3ms horizontally.)

**Fig 4:**
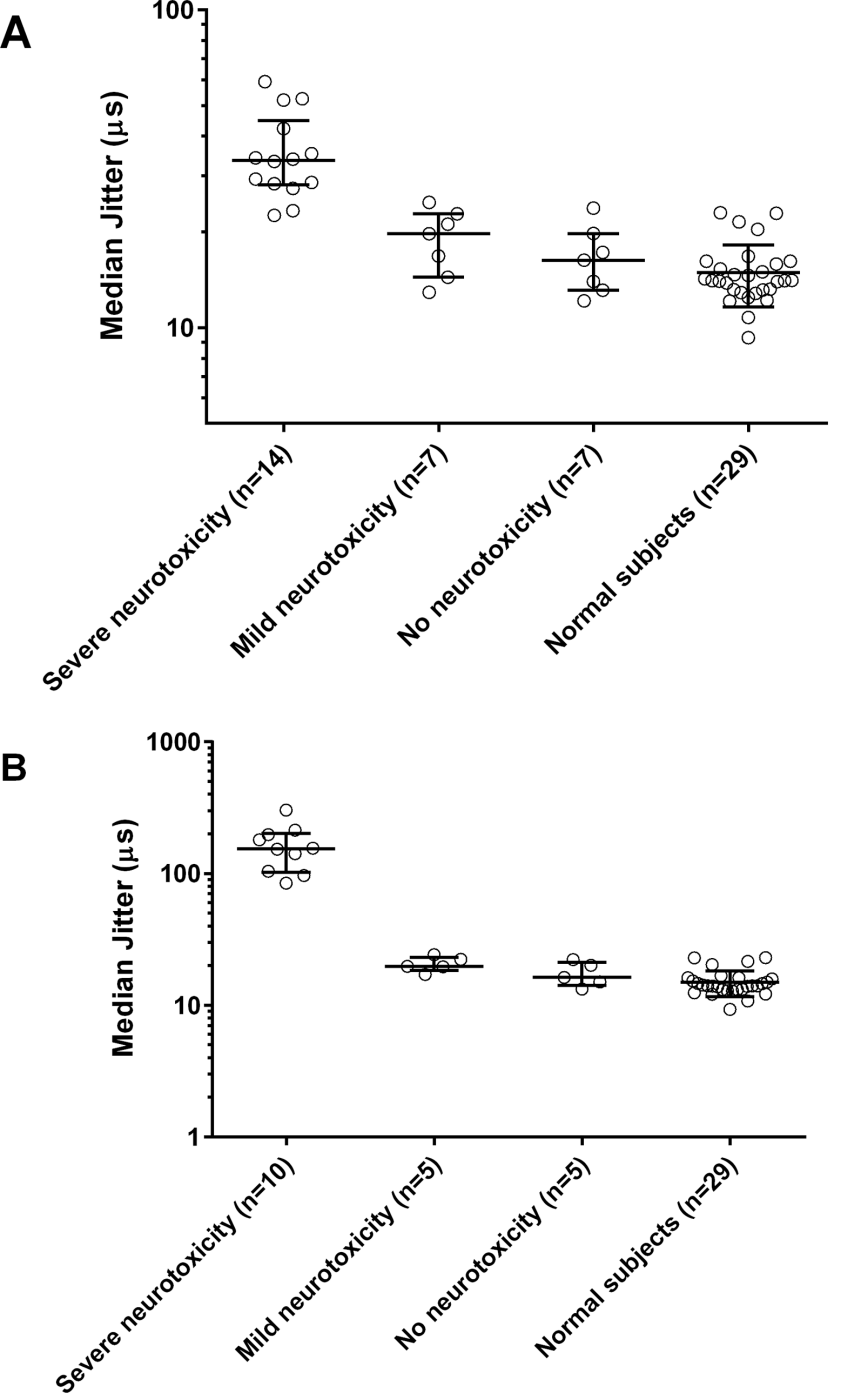
sfEMG jitter comparison across no, mild and severe neurotoxicity groups. Scatter plots of the median MCD in jitter of the three groups of patients with common krait bites on admission **(A)** and 6–12 hours after the bite **(B)** compared to the median MCD in jitter values of normal subjects. Median and interquartile range is shown for each group in the graph. On both occasions, the severe neurotoxicity group has significantly high median jitter compared to the normal subjects (P<0.0001; one-way ANOVA followed by multiple comparison test).

sfEMG were undertaken in seven non-envenomed patients, seven with mild neurotoxicity and ten with severe neurotoxicity. On admission, all patients with no neurotoxicity or mild neurotoxicity (n = 14) had a median MCD in jitter on sfEMG similar to normal controls (Figs 3A and 4A). Two patients with mild neurotoxicity had neuromuscular blockade in 3% and 12% of fibres respectively. The other patients had no neuromuscular block. Jitter was not markedly increased and no blocks were seen in the no neurotoxicity and mild neurotoxicity groups 6–12h post-bite (n = 5, n = 5; Fig 4B). The median MCD was also not different from normal on discharge, at 6 weeks and at 6 to 9 months in all patients in these two groups.

**Fig 5:**
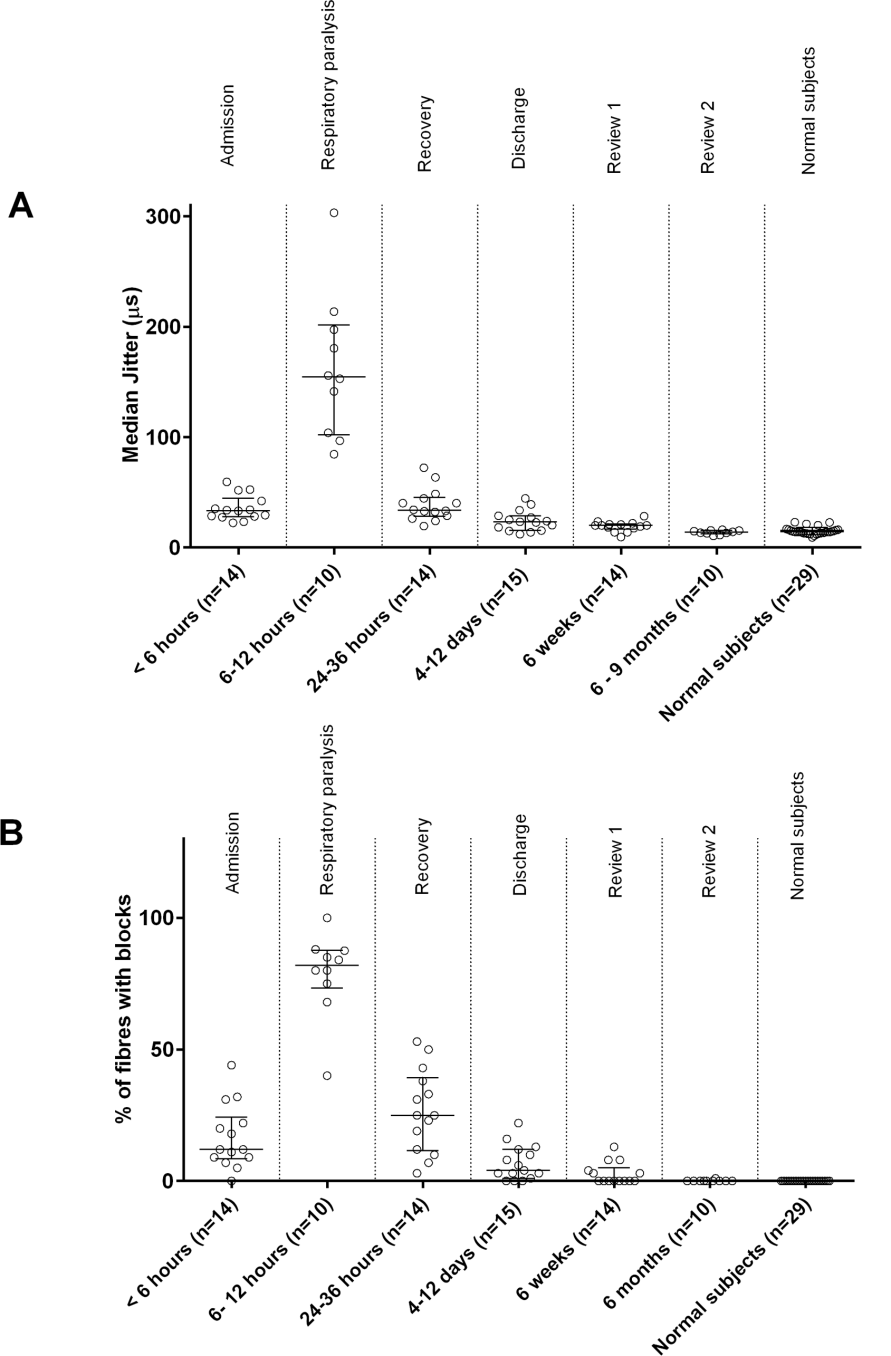
Time related change of sfEMG jitter and blocks in patients developed severe neurotoxicity. **A,** Scatter plots showing the time related change of the median MCD in jitter of the 17 patients with severe neurotoxicity, compared to normal subjects. Note the high median of the MCD in jitter values seen even at 6 weeks after the snakebite, compared to the normal subjects; **B,** scatter plots showing time related changes in the percentage of recorded fibres with neuromuscular blocks in these patients at the same times. Neuromuscular blocks are still present 6 weeks after the snakebite.

All patients with severe neurotoxicity had a high median MCD in jitter compared to normal, with most having neuromuscular blockade on admission (Figs 3B and 4A). At 6–12h post-bite, jitter was markedly increased and there was increased neuromuscular block in recorded fibres (Figs 4B, 5A and 5B). In two patients who had severe neurotoxicity, sfEMG recordings showed no response during this period (9 and 10.5h post-bite respectively), indicating complete neuromuscular blockade. Eleven patients received atracurium for rapid sequence intubation. The post-intubation sfEMG for these patients was done a median of 5.8h (4.5–23.2h) after receiving the atracurium dose.

In all patients with severe neurotoxicity, sfEMG24–36h post-bite showed improved jitter (decreased median MCD) and decreased blocks compared to 6–12h post-bite (Fig 5A and 5B). On discharge, the median MCD in jitter on the sfEMG was still abnormally high in 11 patients. Of these, three had 3 to 10% fibres with blocks (10–12 days post-bite). At 6 weeks, seven of the fourteen patients reviewed still had high MCD jitter values (>22.6μs) and six of them had blocks in 3 to 13% of the recorded fibres. At 6 to 9 months, ten of these patients were reviewed and all had normal jitter with no blocks recorded.

### Measurement of venom concentrations

**Fig 6:**
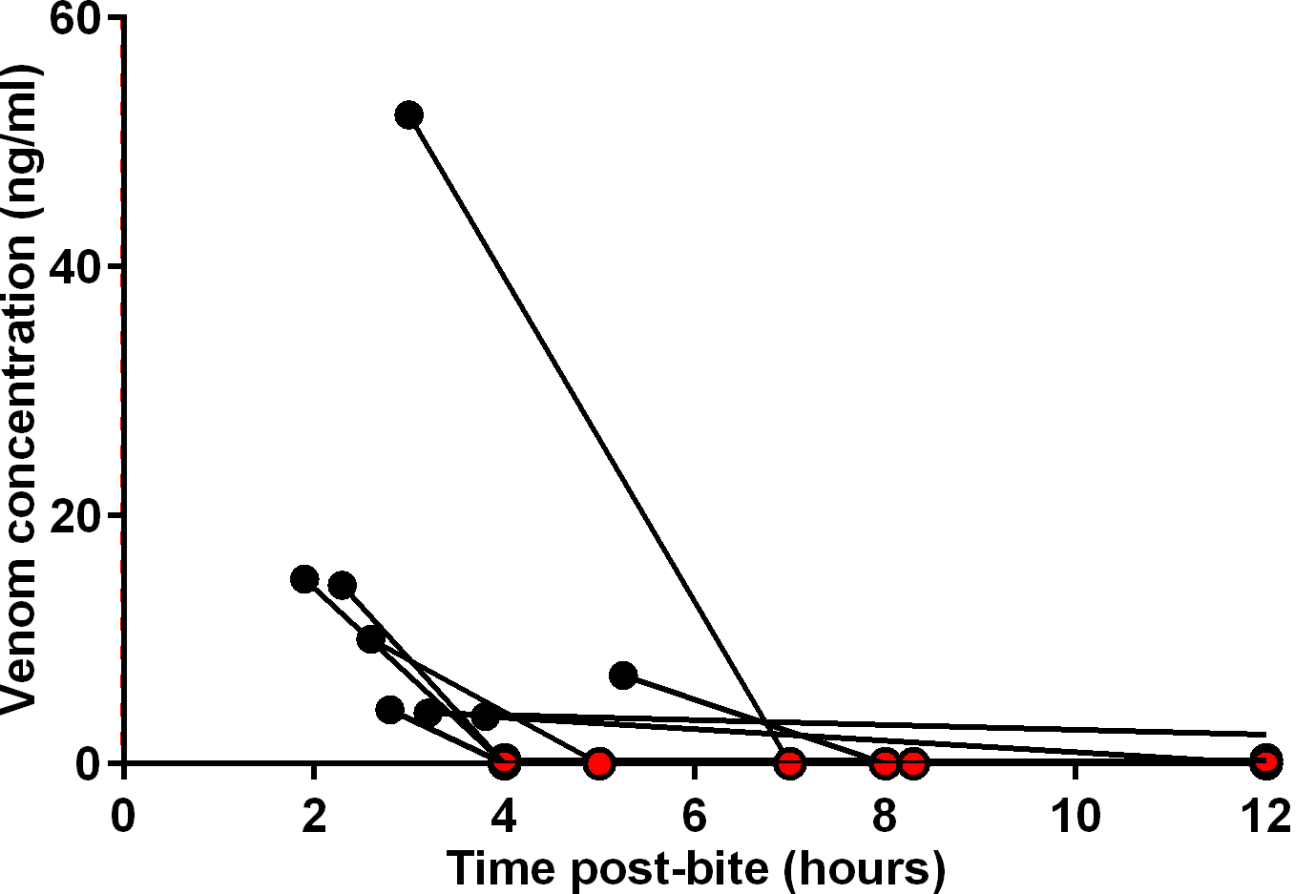
Serum venom concentrations of 9 patients with pre-antivenom blood samples, in relation to the time of initiation of the antivenom therapy. Pre-antivenom venom concentrations are indicated by filled black circles and post-antivenom concentrations by filled red circles.

Krait venom was not detectable in the eight non-envenomed patients. Of the six patients with mild neurotoxicity who received antivenom, pre-antivenom blood samples of four patients were available but no venom was detected in any. The two patients with mild neurotoxicity who did not receive antivenom had no detectable venom in blood. Nine of the 17 patients with severe neurotoxicity had pre-antivenom blood samples available, and krait venom was detected in eight (0.3 to 52.2ng/ml) (Fig 6). None of the post-antivenom samples had detectable free venom, indicating rapid and persistent binding of venom by antivenom (Fig 6).

### Serum creatine kinase concentrations

Serum samples were available in 32 of the 33 study participants and had a median creatine kinase concentration of 43 U/L (8 to 274 U/L). There were no significant differences in the creatine kinase concentrations between the mild, moderate and severely envenomed groups.

## Discussion

In this cohort of patients with definite common krait envenoming, about half developed life-threatening neuromuscular paralysis that did not appear to be prevented by or respond to antivenom treatment. Patients who had sfEMG performed had increased jitter and increased neuromuscular block that correlated with the clinical severity. The neurophysiological abnormalities improved in line with clinical recovery but were still abnormal 6 weeks after the bite, despite the patients being clinically normal. The prolonged high jitter during the recovery phase may represent immaturity of the motor nerve terminals undergoing the re-innervation process. Excepting the three patients who were intubated due to antivenom reactions, all other patients were intubated due to bulbar weakness and/or respiratory paralysis, which developed within 7h of the bite. This demonstrates that severe neuromuscular paralysis develops rapidly. Based on this finding it appears that patients who do not develop bulbar weakness or respiratory paralysis within 12h of the bite, are highly unlikely to develop severe paralysis. These figures are largely in agreement with previous reports from Sri Lanka[7,10].

Although krait venoms contain both pre-synaptic neurotoxins (β-bungarotoxins) and post-synaptic neurotoxins (α-bungarotoxins), it is generally accepted that the pre-synaptic neurotoxins are more important in human envenoming[24,25]. Presynaptic neurotoxins cause irreversible injury. For example, the major pre-synaptic neurotoxin from the Chinese many banded krait (*Bunragus multicinctus*), β-bungarotoxin, causes depletion of synaptic vesicles followed by destruction of the motor nerve terminals in the isolated mouse phrenic nerve-hemi-diaphragm preparation and the soleus muscle of the mouse hind limbs[12]. Common krait venom contains β_1_–β_5_-caerulotoxins, a group of toxins similar to β-bungarotoxin, that are most likely responsible for the paralysis in common krait bites.[26]

Ultrastructural damage and functional injury caused by β-bungarotoxin to the motor nerve terminals recovers over about 7 days[24]. In our study, clinically detectable neurotoxicity resolved 4 to 12 days post-bite, but subclinical neuromuscular dysfunction remained for at least 6 weeks in some patients. This may be due to some motor nerve terminals taking longer to fully recover and re-innervate muscle, and the already re-innervated muscle fibres compensating for recovering fibres. sfEMG directly measures transmission across the neuromuscular junction by measuring security of transmission to individual muscle fibres, so is more sensitive than other neurophysiological investigations and likely to detect abnormal neurotransmission in recovering fibres. Therefore, sfEMG measurements are likely to give a more accurate measure of the recovery time of neuromuscular dysfunction in snake envenoming.

One previous study reported abnormalities in nerve conduction one year after the snake bite, particularly in patients presumed to be bitten by elapids[27]. However, this study by Bell et al. only reported nerve conduction studies one year post-bite, with no comparison at the time of the bite. In addition, these nerve conduction studies are not as sensitive as sfEMG, making it difficult to interpret their results. sfEMG recordings in our study demonstrate the complete recovery of neuromuscular function at 6 months, even in the patients who had severe neuromuscular dysfunction within 24h post-bite, suggesting the findings of Bell et al. one year post-bite are unlikely to be related to the snake bite.

In all 17 patients with severe neuromuscular paralysis, the peak or most severe effects, both clinical and neurophysiological, were observed during the first 24h after the bite. Patients then began to recover during day two, with considerable improvement in both neuromuscular jitter and neuromuscular block compared to day one. In contrast, animal experiments show that the initiation of re-innervation with motor nerve terminal sprouting occurs three to five days after venom inoculation [12,25]. The reason for the more rapid and marked improvement on the second day in humans is unclear. One possible reason is that those muscle fibres of the orbicularis oculi which are already denervated due to toxin induced injury by the second day, fail to produce any response with electrical stimulation. This means they are no longer part of the sampled fibres in the sfEMG recording. Motor nerve terminals that were less damaged are then the ones that make up the majority of the sampled fibres falsely improving the measurement. Although we are unable to exclude this on sfEMG, there was also clinical recovery in patients during the second day, based on the re-appearance of deep tendon reflexes and the plantar reflex. Further investigation is required to understand the pathophysiological basis of these observations in human recovery.

Serum venom concentrations depend on multiple factors including the venom dose delivered, the rate of venom absorption and therefore the time post-bite, individual patient factors that affect the pharmacokinetics of the venom (e.g. effect of patient size/weight, renal function) and the sensitivity of the assay. Kraits inject very small amounts of venom during their bite, explaining the low venom concentrations in our study and previous studies.[9,28] The eight patients who had no detectable venom in blood and had no features of envenoming, were ‘dry bites’, as previously seen among patients with common krait bites[7,9]. The absence of detectable venom in patients with mild neurotoxicity is more difficult to explain. The most likely explanation is that the assay is not sensitive enough to detect the small amount of such a potent elapid venom that can cause minor toxicity. A similar phenomena is seen with Australian brown snake (*Pseudonaja* spp) venom where < 0.2ng/mL can result in a mild coagulopathy [29]. An additional explanation is that this is due to late sampling times after the peak venom concentrations, when venom has distributed out of the central compartment. Either way these patients are likely to have had small venom doses from the bite. This is also the likely explanation for the two patients with mild neurotoxicity who did not receive antivenom and did not worsen. In the severe neurotoxicity group, there was also large variations in the venom concentrations (Fig 6).

Presynaptic neurotoxins cause irreversible nerve injury, so neurotoxicity is expected not to respond to antivenom once it has developed[24]. Despite most patients receiving early antivenom and antivenom rapidly clearing free venom in blood, the paralysis worsened and required mechanical ventilation in all 17 patients for several days. In the mildly neurotoxic patients one patient progressed despite antivenom and two patients who did not receive antivenom had similar outcomes to those receiving antivenom. Antivenom cannot reverse neuromuscular injury and recovery occurs through the natural nerve terminal repair[24,25]. These results demonstrate that Indian polyvalent antivenom is efficacious (binds venom) but is not effective for common krait envenoming in Sri Lanka, because of the irreversibility of the pre-synaptic neurotoxicity.

Antivenom was able to clear circulating free venom, so given early enough antivenom may still be beneficial in preventing progression of neuromuscular dysfunction. This has been demonstrated in studies of Papuan taipan bites where early antivenom (<6h post-bite) reduced the number of patients requiring intubation[17]. Unfortunately, the majority of patients (19/23) who received antivenom in our study developed acute adverse reactions, including some with life threatening anaphylaxis. Therefore, the safety and benefits of antivenom need to be carefully weighed up along with the clinical status of the patient, before deciding on antivenom therapy.

The majority of patients in this study reached a primary care centre early, but because of concerns about antivenom reactions, antivenom was not usually administered prior to transfer to the study hospital. If Indian polyvalent antivenom had a lower reaction rate, this would encourage primary care doctors to administer antivenom as early as possible, and before transferring them to tertiary care hospital. Such an approach would help prevent neurotoxicity in the majority of cases, without risk of life-threatening adverse reactions.

Although generalized myalgia and muscle tenderness were observed in some patients, the normal serum creatine kinase concentrations in patients is consistent with common krait envenoming not causing myotoxicity. Mildly elevated serum myoglobin levels were previously reported in one envenomed krait patient in Sri Lanka,[28] but serum myoglobin is not a very specific marker of muscle injury. Myotoxicity has been reported in envenoming by other krait species, including *B*. *niger* [30], *B*. *multicinctus* [31] and *B*. *candidus* [32]. However, in the study of *B*. *candidus* there were only mild elevations of creatine kinase, and the study of *B*. *multicinctus* only reports myalgia.

Coma has been previously reported in common krait envenoming [7,33]. In one study, two patients with deep coma were reported to have electroencephalogram abnormalities, abnormal brain stem visual and auditory evoked potentials, leading to the conclusion that krait venom can cause cortical and brain stem effects [33]. However peptide and protein toxins are unlikely to cross the blood brain barrier making this theoretically unlikely. In the present study, one patient with severe paralysis had deep coma, absent brainstem reflexes and no sfEMG recordings. Interestingly, there was a period of time when the patient had absent brain stem reflexes but some motor function, suggesting that the patient was more likely to have had severe paralysis mimicking coma, rather than coma itself. Similar observations have previously been made in snakebite patients in India [34–37]. The altered consciousness observed in three patients on admission was most likely due to hypoxia secondary to respiratory muscle paralysis, rather than any direct central effect of the venom.

sfEMG jitter results can be influenced by pre-existing medical conditions that affect the peripheral nervous system, such as myasthenia gravis, diabetes mellitus and leprosy. None of the patients in this study had a history of any of these conditions. Two-thirds of the patients were farmers who may have had pre-existing neurotransmission abnormalities secondary to chronic exposure to organophosphates. However, we did not see a difference in the jitter values of the present cohort of patients at 6 months compared to the normal subjects, so this is unlikely.

A limitation of the study was that sfEMG was only performed on the orbicularis oculi muscle. This was done because it is one of the muscles affected earliest in snake bite paralysis and it is convenient to access. The neuromuscular jitter and blocking correlated well with the clinical picture indicating that this muscle is likely to be representative of the neurophysiology of the neuromuscular paralysis in snake envenomed humans. Another limitation of the study was that the recovery of certain muscles, e.g. neck extensors, buccinator, bulbar muscles, could not be assessed while patients were intubated. In addition, while the patients were sedated, assessment of the power of voluntary contractions of muscles was not possible. Hence the exact sequence of muscle involvement, particularly during the recovery, could not be documented in some patients. Finally, it is important to consider the risk of sfEMG in patients with coagulopathy.

Our study highlights the usefulness of sfEMG as a biomarker, particularly as a research tool on snakebite neurotoxicity. The sfEMG and clinical findings suggest that recovery occurs more rapidly than expected, based on animal studies, and further work is required to explain this. The study confirmed that antivenom did not reverse neurotoxicity and that if antivenom is going to prevent neurotoxicity, it must be given much earlier, prior to development of any neurotoxic effects. A placebo controlled trial is required to determine if antivenom hastens the recovery in established neurotoxicity.
